# Real-time RT–PCR detection of disseminated tumour cells in bone marrow has superior prognostic significance in comparison with circulating tumour cells in patients with breast cancer

**DOI:** 10.1038/sj.bjc.6602985

**Published:** 2006-02-21

**Authors:** I H Benoy, H Elst, M Philips, H Wuyts, P Van Dam, S Scharpé, E Van Marck, P B Vermeulen, L Y Dirix

**Affiliations:** 1 Translational Cancer Research Group Antwerp, Department of Pathology University of Antwerp/University Hospital Antwerp, Edegem 2650, Belgium; 2 Translational Cancer Research Group Antwerp, Oncology Centre, General Hospital Sint-Augustinus, Oosterveldlaan 24, Wilrijk 2610, Belgium; 3 Medical Biochemistry, University of Antwerp, Wilrijk 2610, Belgium

**Keywords:** breast cancer, disseminated epithelial cells, cytokeratin-19, mammaglobin, RT–PCR

## Abstract

This study assessed the ability of real-time reverse transcription–polymerase chain reaction (RT–PCR) analysis to detect disseminated epithelial cells (DEC) in peripheral blood (PB) and bone marrow (BM) of patients with breast cancer (BC). Detection of DEC in BM is an obvious choice in BC, but blood sampling is more convenient. The aim of this study was to evaluate whether the detection of DEC in either PB or BM predicts overall survival (OS). Peripheral blood and BM samples were collected from 148 patients with primary (stage M0, *n*=116/78%) and metastatic (stage M+, *n*=32/21%) BC before the initiation of any local or systemic treatment. Peripheral blood of healthy volunteers and BM of patients with a nonmalignant breast lesion or a haematological malignancy served as the control group. Disseminated epithelial cells was detected by measuring relative gene expression (RGE) for cytokeratin-19 (CK-19) and mammaglobin (MAM), using a quantitative RT–PCR detection method. The mean follow-up time was 786 days (+/− 487). Kaplan–Meier analysis was used for predicting OS. By taking the 95 percentile of the RGE of CK-19 (BM: 26.3 and PB: 58.7) of the control group as cutoff, elevated CK-19 expression was detected in 42 (28%) BM samples and in 22 (15%) PB samples. Mammaglobin expression was elevated in 20% (both PB and BM) of the patients with BC. There was a 68% (CK-19) and 75% (MAM) concordance between PB and BM samples when classifying the results as either positive or negative. Patients with an elevated CK-19 or MAM expression in the BM had a worse prognosis than patients without elevated expression levels (OS: log-rank test, *P*=0.0045 (CK-19) and *P*=0.025 (MAM)). For PB survival analysis, no statistical significant difference was observed between patients with or without elevated CK-19 or MAM expression (OS: log-rank test, *P*=0.551 (CK-19) and *P*=0.329 (MAM)). Separate analyses of the M0 and M+ patients revealed a marked difference in OS according to the BM CK-19 or MAM status in the M+ patient group, but in the M0 group, only MAM expression was a prognostic marker for OS. Disseminated epithelial cells, measured as elevated CK-19 or MAM mRNA expression, could be detected in both PB and BM of patients with BC. Only the presence of DEC in BM was highly predictive for OS. The occurrence of DEC in the BM is probably less time-dependent and may act as a filter for circulating BC cells. The use of either larger volumes of PB or performing an enrichment step for circulating tumour in blood cells might improve these results.

Breast cancer (BC) remains an important public health problem. In the 21st century, one out of nine women will develop BC. Most patients present with stage I or II disease and at least up to 30–40% of these patients will develop recurrent disease. These patients are considered as having disseminated cancer cells at the time of local treatment. Current guidelines for the adjuvant treatment in lymph-node-negative BC patients have been shown to result in overtreatment, with inherent disadvantages and even health risks (Shapiro and Recht, 2001; Goldhirsh and Senn, 2003). Accurate staging of patients diagnosed with BC is important to determine the extent of disease and to plan appropriate therapies.

Molecular diagnostics have, for the first time, been integrated in the revised tumour node metastasis (TNM) staging system for BC in order to collect data on minimal disease in lymph nodes that may affect treatment in the future (Singletary *et al*, 2002). Analogous to the staging procedures in haematological malignancies, detection of minimal disease in bone marrow (BM), defined as single or clumped disseminated cancer cells, has been suggested to be a more direct approach to select the metastasis-prone patients among the ‘good prognosis group’, based on the TNM staging (Braun and Pantel, 1998). Numerous studies have elaborated on this concept by detecting disseminated epithelial cells (DEC), or transcripts of supposedly specific markers for epithelial cells, in BM aspirates (Diel *et al*, 1996; Braun *et al*, 2000; Janni *et al*, 2000; Gerber *et al*, 2001; Wiedswang *et al*, 2003). In these studies, the BM status is considered as a mirror for the efficacy of the metastatic process throughout the body, similar to detection of cancer cells in lymph nodes. Also, the prognostic relevance of DECs in BM is clearly demonstrated by a number of large studies (Braun *et al*, 2000, 2005; Weinschenker *et al*, 2004). The vast majority of these studies used immunocytochemistry for cell detection. Currently, detection by polymerase chain reaction (PCR) techniques is much less validated.

The detection of tumour cells in BM is an obvious choice in BC, since a majority of patients will develop bone metastases once dissemination has been detected clinically (Rubens, 1998). Although BM aspiration is an acknowledged clinical method, it remains cumbersome, especially if repetitive examinations are considered. Peripheral blood (PB) is an other organ to evaluate a patient for the presence of disseminated epithelial cancer cells, although tumour cells in blood and tumour cells in BM do not necessarily have the same metastatic potential. Repetitive sampling of PB is accepted.

In this study, we attempted to quantify transcripts of one marker considered relatively specific for epithelial cells, cytokeratin-19 (CK-19), and of a more breast-tissue-specific cell marker, mammaglobin (MAM), in both the PB and BM of patients with BC using quantitative real-time reverse transcription (RT)–PCR according to the Taqman methodology. The hypothesis was that the use of a quantitative technique would allow for the discrimination between low amounts of epithelial-cell-related present in the blood or BM of healthy individuals and the presumably elevated amounts of tumour-cell-related transcripts present in the blood or BM of advanced cancer patients, both resulting in a ‘positive’ signal with nonquantitative techniques.

Cytokeratin-19 was chosen as it is expressed in the majority of BCs (Bartek *et al*, 1986) and has been extensively used in studies in this area (Slade *et al*, 1999; Smith *et al*, 2000; Aerts *et al*, 2001; Stathopoulou *et al*, 2003; Benoy *et al*, 2004; Ring *et al*, 2005). Mammaglobin gene expression has previously been used to detect circulating BC micometastases with no false-positives in the control population (Zach *et al*, 1999; Grunewald *et al*, 2000; Suchy *et al*, 2000; Leone *et al*, 2001; Silva *et al*, 2002; Ring *et al*, 2005).

The aim of this study was to compare directly the detection rate of DEC in blood and BM by a sensitive and specific real-time RT–PCR method and to clarify their prognostic value. We investigated the possibility of replacing BM aspiration by PB sampling for the detection of isolated tumour cells. Therefore, this study is conducted on simultaneously acquired blood and BM samples from 148 patients with BC with stage I to IV disease before the initiation of any local or systemic treatment.

## PATIENTS AND METHODS

### Patients and sample handling

Peripheral blood samples and BM aspirates were obtained from 148 patients with BC (age: 27–88 years), 116 patients with primary disease (M0, 78%) and 32 patients with metastatic disease (M+, 21%). Samples were collected before surgery or implementation of therapy and the M+ patients had no previous treatment for metastatic disease. All patients were treated in the General Hospital Sint-Augustinus and follow-up data were obtained prospectively. Clinical and histopathological data, therapy, relapse and survival information from each patient were entered in a database. The mean follow-up time was 786 days (s.d.: 487, median 855). All analysis were conducted blind of the patients’ clinicopathological status.

Peripheral blood samples from 37 healthy female volunteers (age: 21–80 years) served as controls. A measure of 9 ml of PB was stored in EDTA-containing tubes (Becton Dickinson, Vacutainer system, Belgium) and processed within 2 h after sampling. To avoid epithelial contamination by the skin during venipuncture, the first 8 ml of blood was discarded.

Bone marrow aspirates were taken under locale or general anaesthesia. Bone marrow aspirates from 13 patients with a nonmalignant breast lesion or a haematological malignancy served as control patients (age: 25–79 years). A measure of 9 ml of BM was aspirated from the posterior iliac crest into syringes containing 5000 IU heparin as anticoagulant. To avoid epithelial contamination during aspiration, a small incision was made in the skin and the first 2 ml of BM was discarded. The BM aspirate was transported to the lab within 30 min. Mononuclear cells (MNC) were isolated by density-gradient centrifugation through Ficoll–Hypaque (Amersham Pharmacia Biotech, Sweden) and washed twice with PBS. After centrifugation, the cell pellet was resuspended in a guanidine isothiocyanate-containing buffer.

The patients were divided into two groups: without overt metastasis (M0) and with metastasis (M+). The study protocol was approved by the ethical committees of the Faculty of Medicine, University of Antwerp, and of the General Hospital Sint-Augustinus. All patients and volunteers signed a written informed consent.

### RNA isolation and cDNA synthesis

Total RNA was isolated from the blood, using the Qiagen Rneasy Total Blood RNA Kit. From the BM MNC, total RNA was extracted with the RNeasy midi kit (Qiagen, Germany). The exact volume of blood and BM (range PB: 8–9 ml; BM: 4–9 ml), used for RNA extraction and the exact elution volume (range 150–600 *μ*l) were documented to enable the calculation of target mRNA concentration afterwards.

The concentration of RNA was measured spectrophotometrically. All samples had an OD 260/280 nm ratio >1.8. The RNA integrity was tested on the Agilent 2100 Bioanalyzer. Only samples lacking degradation on the electropherogram and with a good 28S/18S ratio were analysed.

For the generation of first-strand cDNA, 2 *μ*g of total RNA was reverse-transcribed in a final volume of 100 *μ*l with the High-Capacity cDNA Archive Kit (Applied Biosystems, The Netherlands).

### PCR amplification

CK-19 and MAM mRNA expression was measured as described before (Benoy *et al*, 2004). cDNA-specific CK-19 and hMAM Taqman™ primer and probe sets were developed using Primer Express® software. To avoid amplification of contaminating genomic DNA, primers and probes were placed on different exons. The forward primer of CK-19 (CCCGCGACTACAGCCACTA) is situated on exon 1, the probe (FAM-ACCATTGAGAACTCCAGGATTGTCCTGCA-TAMRA) on exon 2 and the reverse primer (CTCATGCGCAGAGCCTGTT) on exon 3. Reverse transcription–polymerase chain reaction using this primer set resulted in a 163 bp fragment. For hMAM, the forward primer (ATGAAGTTGCTGATGGTCCTCAT) and the probe (FAM-CGGCCCTCTCCCAGCACTGC-TAMRA) are located on exon 1 and the reverse primer (GTCTTAGACACTTGTGGATTGATTGTCT) on exon 2. The hMAM amplicon consists of 119 bp. The nucleotide sequences of the primers and probes were checked for their specificity in the NCBI BLAST® database.

All PCR reactions were performed on the ABI Prism 7700 Sequence Detection System (Applied Biosystems) using the fluorescent Taqman methodology. The PCR cycle at which the fluorescence arises above the background signal is called the cycle threshold (*C*_t_).

A measure of 10 *μ*l of the reverse transcription volume was used for each PCR reaction in a total volume of 50 *μ*l. Commercially available primers and probe for *β*-actin mRNA were used for normalisation (Applied Biosystems). This probe is labelled with a VIC dye, and to avoid competition in the multiplex PCR reaction tube, the concentrations of the primers are limited.

The CK-19 and MAM mRNA quantities were analysed in triplicate and mean *C*_t_ levels were used for further analyses. Results were normalised against *β*-actin and expressed in relation to a calibrator sample. As described by Livak and Schmittgen (2001), results per PCR reaction were expressed as relative gene expression (RGE), using the delta–delta *C*_t_ method. The calibrator was produced from the blood of a healthy volunteer spiked with 5 MDA-MB361 cells per 10^6^ blood cells. The calibrator was given an RGE value of 100. As we are only interested in the amount of CK-19 or MAM expression per ml of sample, regardless of the amount of white blood cells, RGE per PCR reaction was normalised according to the following equation: 
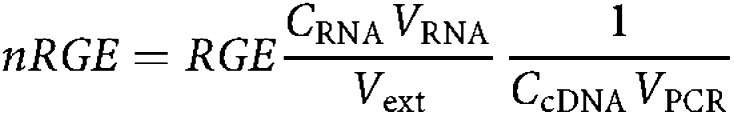
 where *nRGE* is the normalised *RGE* expressed as relative target concentration per ml PB or BM (RGE per ml sample); *RGE* is the relative gene expression of target determined by sequence detector per PCR reaction; *C*_RNA_ is the concentration of totalRNA extracted per sample; *V*_RNA_ is the elution volume of totalRNA obtained after extraction, typically 300 *μ*l per Qiagen RNeasy Midi Extraction; *V*_ext_ is the volume of PB or BM extracted, typically 9 ml; *C*_cDNA_ is the concentration cDNA, typically 2 *μ*g/100 *μ*l; and *V*_PCR_ is the volume of cDNA solution used for PCR amplification, typically 10 *μ*l.

### Statistics

Data were analysed using the statistical software package SPSS 12.0. The Mann–Whitney *U*-test was used to validate differences in gene expression of CK-19 and MAM between the control and patient populations. Correlation analyses were validated by the Spearman rho correlation test for continuous nonparametric variables and by the kappa test for categorical variables (Landis and Koch, 1977). Differences in positivity rates between blood and BM samples were assessed using the McNemar test. The *χ*^2^ test and linear-by-linear test were used to assess the relation between patient characteristics and rates of positive samples.

Overall survival (OS) was calculated from the day of diagnosis (sample take) until death or last follow-up, regardless of the cause of death. Distance disease-free survival (DDFS) was calculated for the M0 group from the day of diagnosis until relapse. Survival curves were calculated for each group with Kaplan–Meier estimates and compared with the log-rank test. Cox's proportional-hazards regression was used for univariate and multivariate (stepwise backward elimination) analysis of prognostic impact of relevant variables. *P*-values smaller than 0.05 were considered statistically significant.

## RESULTS

### CK-19 and MAM mRNA expression in PB samples of healthy volunteers and patients with BC

To determine the cutoff point for CK-19 mRNA and MAM mRNA expression in blood, RGE was determined in 37 blood samples of healthy volunteers (Table 1). In all these blood samples, CK-19 mRNA was measurable with a median nRGE of 26.5 (range: 0.4–104). The cutoff value, determined as the 95 percentile of the CK-19 nRGE values of healthy volunteers, was 58.7. Mammaglobin (MAM) expression was measurable in four of the 37 control blood samples. The cutoff level was set as 0.092 (95 percentile of the normal MAM values).

Results for nRGE of CK-19 and MAM mRNA expression in PB samples of patients with BC are summarised in Table 1.

**Figure 1:**
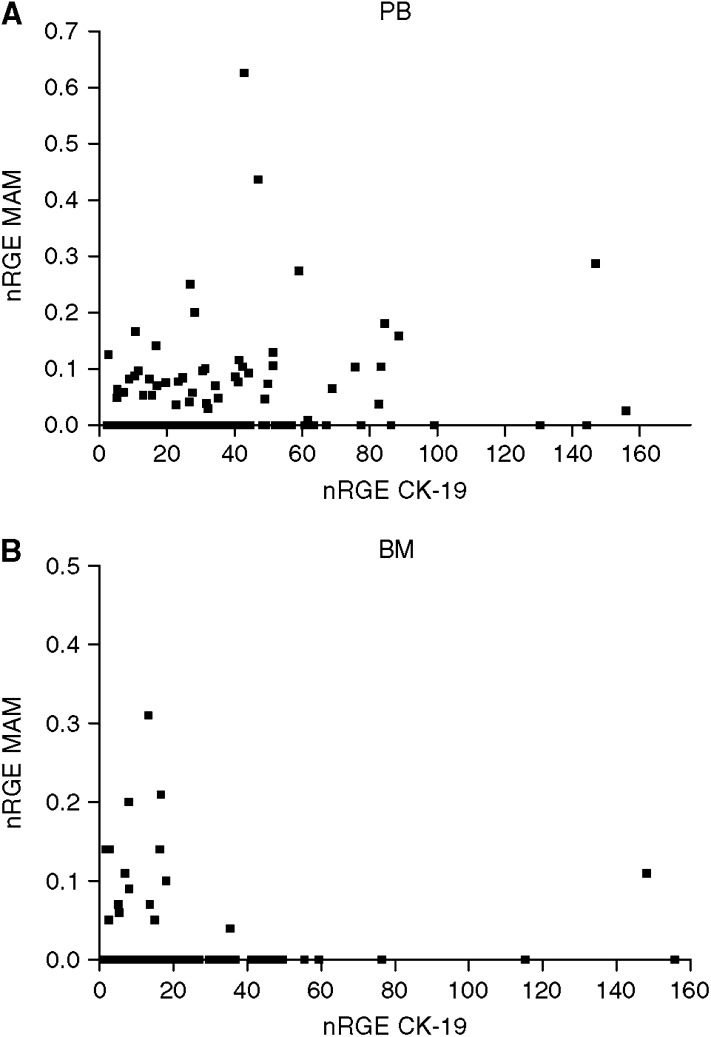
Correlation between CK-19 and MAM relative gene expression (nRGE) in (**A**) peripheral blood (PB) and (**B**) bone marrow (BM).

The correlation between CK-19 and MAM expression is illustrated in Figure 1A (Spearman rho=0.199, *P*=0.015). Nine patients had an elevated expression of both CK-19 and MAM in their blood, 13 samples had only an elevated CK-19 expression and a normal MAM expression, whereas 20 patients showed an elevated MAM expression with a normal CK-19 expression. In all, 106 patients had both a normal CK-19 and MAM expression.

Significant differences in expression level of MAM were observed between negative control samples, patients with nonmetastatic BC and patients with metastatic BC. For CK-19, only a difference in expression level was observed between the negative control group and the M+ group (Mann–Whitney U-test; Table 1).

### CK-19 and MAM mRNA expression in BM

In none of the 13 negative control BM samples MAM expression was measurable by RT–PCR. On the other hand, CK-19 mRNA could be quantified in all control samples, with a median RGE of 9.94 (range 4–36) (Table 1). With the 95 percentile from the CK-19 RGE (26.3) of the negative control group as cutoff, 15 of the 32 (47%) BM aspirates from the M+ patients had an increased CK-19 expression. MAM expression was measurable in 12 of the 32 (38%) samples. Results for the M0 group are presented in Table 1.

The correlation between CK-19 and MAM expression is illustrated in Figure 1B (Spearman rho=0.254, *P*=0.0019). In total, 14 patients had an elevated expression of both CK-19 and MAM in their BM, 28 samples had only an elevated CK-19 expression and a normal MAM expression, whereas 16 patients showed an elevated MAM expression with a normal CK-19 expression. A total of 90 patients had both a normal CK-19 and MAM expression.

A significant difference in CK-19 and MAM mRNA expression in BM is observed between negative control samples or patients with operable BC and patients with disseminated disease (Table 1).

### Concordance of CK-19 and MAM expression in blood and BM samples

A strong correlation was found for CK-19 and MAM nRGE in blood and BM samples (CK-19: Spearman rho=0.3, *P*=0.0003, 95% confidence interval (CI)=0.14–0.44/MAM: Spearman rho=0.25, *P*=0.024, 95% CI=0.09–0.40). When the different patient groups were analysed separately, the correlation between blood and BM was confirmed (except for MAM expression in the M0 group) (data not shown).

In only 19% (eight of 42) of the patient samples with a BM CK-19 RGE above the cutoff, CK-19 expression was also elevated in the PB sample. On the other hand, 87% (92 of 106) of the patients with normal CK-19 expression in the BM sample also had normal CK-19 expression in the PB (Table 2). Overall, there was 68% (100 of 148) concordance between blood and BM for CK-19 mRNA positivity, with a slight kappa value (0.07). According to the McNemar test, there was a significant difference in positivity between blood and BM CK-19 expression (*P*=0.006).

For MAM expression, a concordance of 75% (111 of 148) was found between blood and BM (Table 2). The kappa value for MAM expression was fair (0.22) and there was no difference in positivity according to the McNemar test (*P*=1).

### Relation of DEC in blood and BM with clinicopathological parameters

Results of the *χ*^2^ test, to assess the relation between patient characteristics and rates of positive samples for the presence of DECs in blood and BM of patients with BC, are presented in Table 3. No relation was found between the presence of DEC in blood or BM and T stage, N stage, oestrogen receptor (ER) hormone receptor status, histology, histological grade or menopausal status. The presence of elevated CK-19 or MAM expression in BM was correlated with PR hormone receptor status.

### DEC and survival

**Figure 2:**
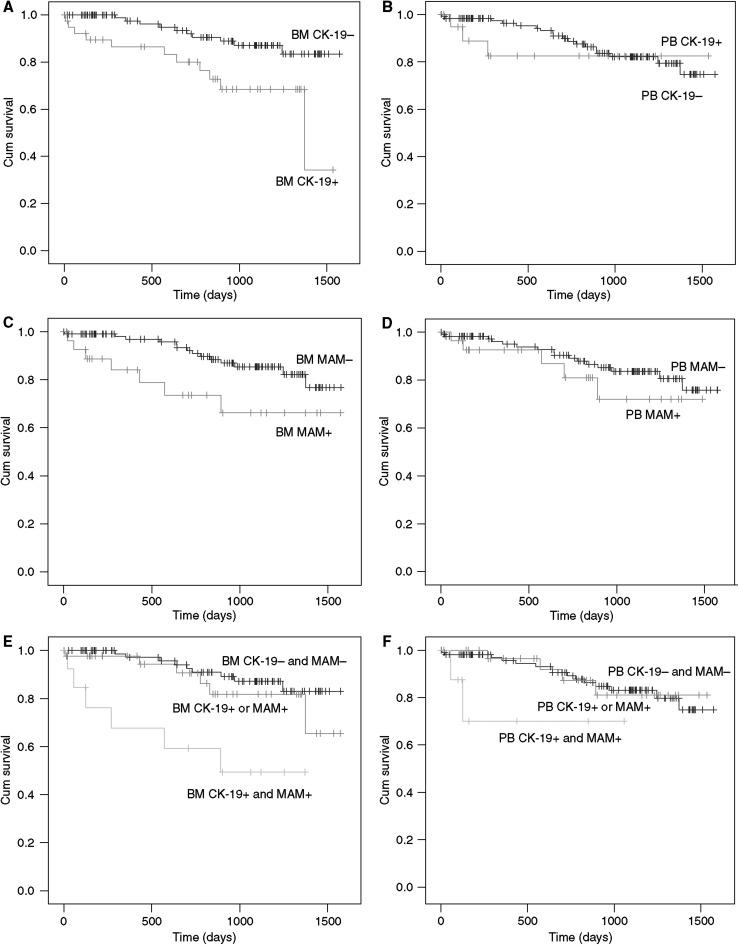
Kaplan–Meier curves of overall survival of patients with breast cancer according to the presence or absence of disseminated epithelial cell in bone marrow (BM+ or BM−) (curves **A**, **C** and **E**) or in peripheral blood (PB+ or PB−) (curves **B**, **D** and **F**) and according to cytokeratin-19 (CK-19) (curves **A**, **B**, **E** and **F**) or mammaglobin expression (MAM) (curves **C**, **D**, **E** and **F**).

During the observation period, 21 of the 148 patients with BC have died. In the BM CK-19+ group, 11 of the 42 patients died, compared with 10 of the 106 BM CK-19− group. When analysing survival data according to the MAM expression in BM, seven of the 30 BM MAM+ patients died, compared with 14 of the 118 BM MAM− patient group (Table 4). Kaplan–Meier survival analysis demonstrates a markedly reduced OS among the BM+ patients (OS: log-rank test, *P*=0.0045 (CK-19) and *P*=0.025 (MAM)) (Figure 2). Among women with an elevated CK-19 mRNA expression in the BM, as compared with those with a normal CK-19 expression, the relative risk of death was 3.26 (95% CI 1.38–7.73, *P*=0.007). The relative risk for patients with elevated MAM mRNA expression in the BM was 2.72 (95% CI 1.10–6.75), compared with those without MAM mRNA expression (Table 4). Patients with an elevated CK-19 and MAM expression (double phenotypes) have the worst prognosis (Figure 2 and Table 4).

In contrast to the BM status, presence of circulating tumour cells in the PB had no impact on the OS of the patient (OS: log-rank test, *P*=0.551 (CK-19) and *P*=0.329 (MAM)).

Separate analyses of the M0 and M+ patients revealed a marked difference in OS according to the BM CK-19 or MAM status in the M+ patient group, but in the M0 group, only MAM expression was a prognostic marker for OS (OS: log-rank test, *P*=0.125 (CK-19) and *P*=0.041 (MAM), *P*=0.035 (double phenotypes)). Survival analyses for DDFS were comparable with OS for these M0 patient group (DDFS: log-rank test, *P*=0.204 (CK-19) and *P*=0.063 (MAM), *P*=0.049 (double phenotypes)).

### Multivariate analysis for OS

Following parameters were evaluated for OS: BM status, PB status, menopausal status, tumour status, lymph node status, metastatic status, hormone receptor status, histological grade, tumour histology and Her2 status. On multivariate analysis, metastatic stage, histological grade and BM CK-19 mRNA expression were the only independent factors of poor prognosis. Results are summarised in Table 5.

## DISCUSSION

This study describes a sensitive method to quantify CK-19 and MAM mRNA expression in blood and BM samples, using real-time RT–PCR and the fluorescent Taqman assay. This mRNA is supposed to be derived from disseminated tumour cells, performing critical steps of the metastatic cascade. In support of this concept are the higher expression levels obtained in the blood and BM of patients with advanced BC in this and other studies (Aerts *et al*, 2001; Stathopoulou *et al*, 2003; Benoy *et al*, 2004; Slade *et al*, 2005).

Two distinct approaches are widely used to detect isolated tumour cells: immunocytochemical (ICC)- and molecular biological-based methods. Immunocytochemical is still the standard method for tumour cell detection, but in the last years, there is a rapid expansion in the application of molecular PCR-based methods to detect isolated tumour cells in haematological fluids. The problem of PCR analysis is the specificity and the reproducibility of the different methods described in the literature. The need for validation of the results in an independent validation cohort is advisable.

CK-19 mRNA expression was measurable in PB of healthy volunteers and in the BM of negative control patients. In order to improve the specificity of the assay – a specificity that is negatively affected by the high sensitivity of the RT–PCR technique – establishing a cutoff was pursued instead of performing a negative immunological preselection or another method to eliminate fractions of nonepithelial blood cells that might express CK-19 at low levels. The rationale was that the latter methods unavoidably induce additional methodological variabilityies. The cutoff value was predefined as the 95th percentile value of CK-19 mRNA expression measured in the control population. The aim was to design a sensitive tool to detect ongoing metastasis, even in patients with operable disease, in order to minimise false-negativity. By adopting more stringent criteria like changing the cutoff to even higher than 95% of the control population (eg maximum value of the control population +2 s.d.), survival analysis for positive BM status will not change. For blood, only one patient will be positive for the presence of circulating tumour cells, which makes survival analysis meaningless.

Also, MAM mRNA expression was determined quantitatively in blood and BM samples from patients with BC. In contrast to the studies of Zach, Suchy, Corradini, Silva and Zehenter (Zach *et al*, 1999; Suchy *et al*, 2000; Corradini *et al*, 2001; Silva *et al*, 2002; Zehentner *et al*, 2004), MAM mRNA could be amplified in four of the 37 PB samples of the healthy control population. Amplification of MAM mRNA in these four blood samples was only measurable in one of the three replicates and with a *C*_T_ value around the detection limit of the PCR reaction (*C*_T_ around 40 cycles) and is the result of the high sensitivity of the methodology. Therefore, analogous to the CK-19 mRNA expression, a cutoff value was calculated.

In our study, we observed higher detection rates of DEC in BM samples (CK-19: 28%) than in PB samples (CK-19: 15%) of patients with BC. This is in agreement with several other studies (Schoenfeld *et al*, 1997; Slade *et al*, 1999; Berois *et al*, 2000; Stathopoulou *et al*, 2002; Ismail *et al*, 2004; Pierga *et al*, 2004), all of whom demonstrate that BM is more likely to be positive than PB for the presence of DEC (Table 4). Cancer cells may be intermittently shed into the bloodstream, which could result in a sampling error if a single-point sampling is evaluated. The occurrence of DEC in the BM of BC patients is probably less time-dependent and may act as a filter for circulating BC cells.

Classifying the results as negative or positive for the presence of DEC, we retained with real-time RT–PCR analysis a concordance between blood and BM samples for CK-19 expression of 68% and for MAM expression of 75%. These results are consistent with the findings of the other groups (Table 6). Statistically, no association is found between CK-19 expression in blood and in that BM. It is not yet clear whether all circulating tumour cells in PB can establish tumours at other sites.

During the observation period with a mean of 786 days, 21 of the 148 patients have died. According to the survival analysis of this group of 148 patients with BC, the presence of DEC in the BM and not in PB is associated with poor prognosis. As the observation period is relatively short when analysing only OS data for the M0 patient group, MAM expression or double phenotypes remain(s) a prognostic marker, but there is a trend towards a significant difference in survival in favour of patients who were CK-19− in the BM.

Our results confirm the survival analyses of the group of Wiedswang *et al* (2005), leading to the conclusion that the detection of DEC in PB is quantitatively markedly different from, and prognostically inferior to, the detection of DEC in BM. Pierga *et al* (2004) concluded that the clinical value of circulating epithelial cells has yet to be established. In their study, the presence of tumour cells in the BM appears to be more predictive of relapse than the presence of tumour cells in PB. The use of larger volumes of PB or performing an enrichment step for circulating tumour cells may be a better alternative. The recent studies of Christofanilli seem to be promising: patients with metastatic BC who have more than five circulating tumour cells after enrichment in 7.5 ml blood have a worse prognosis than patients with less than five cells (Cristofanilli *et al*, 2004, 2005).

No correlation is observed between the presence of DEC in blood or BM and conventional pathological markers such as tumour size, lymph node status, ER hormone receptor status, histology or Her2 status. These results are in agreement with the studies of Braun and Schindlbeck, where established pathological parameters did not predict the presence of ICC-detected CK+ in BM samples (Braun *et al*, 2000; Schindlbeck *et al*, 2004). However, in the meta-analysis of Weinschenker, a direct correlation was observed between ICC BM positivity and the primary tumour's presence with expression of ER, large size and higher histologic grade (Weinschenker *et al*, 2004). In the study of Pierga *et al*, the presence of ICC CK+ cells in blood in patients with operable disease was correlated with negative ER and nodal involvement. The presence of ICC CK+ cells in the BM in these patients was correlated with premenopausal status, Oestrogen-negative tumours and clinical tumour size (Pierga *et al*, 2004). Lack of correlation between expression of MAM mRNA in PB and known prognostic factors for BC was also described by Lin *et al* (2003), who used a conventional qualitative RT–PCR detection method.

In conclusion, we have developed a molecular method to quantify CK-19 mRNA and MAM mRNA in PB and BM with high sensitivity. By adopting a cutoff level of expression, a significant difference between CK-19 mRNA load of the blood and BM of healthy volunteers and of metastatic BC was found.

Bone marrow is more likely to be positive than PB for the presence of DEC and independently predicts OS. The prognostic value of BM involvement is also clearly demonstrated by a number of large studies (Braun *et al*, 2000, 2003, 2005; Gebauer *et al*, 2001; Gerber *et al*, 2001), which is less well established for blood (Cristofanilli *et al*, 2004, 2005). Enrichment of circulating tumour cells may be more promising. As repetitive sampling of blood is more feasible than BM aspiration, detection of circulating epithelial cells in blood would constitute a possible alternative or complement to BM surveillance.

## Figures and Tables

**Table 1:** RT–PCR results for the detection of disseminated epithelial cells in different patient groups

	**PB_nCK^a^**	**PB_nMAM^b^**	**BM_nCK^c^**	**BM_nMAM^d^**
*NC* e				
*N*	37	37	13	13
Median	26.50	0	9.94	0
Minimum	0.37	0	4.12	0
Maximum	103.72	0.216	36.01	0
Cutoff	58.7	0.092	26.3	0
				
*M0* f				
*N*	116	116	116	116
Median	26.4867	0	12.0675	0
Minimum	2.58	0	1.15	0
Maximum	155.94	12.88	231368.22	1171.36
#pos (%)	14 (12)	16 (14)	27 (23)	18 (16)
*P*-value *vs* M+				
				
*M*+^g^				
*N*	32	32	32	32
Median	34.2550	0.0592	24.7800	0
Minimum	2.30	0	2.47	0
Maximum	147.69	182.16	2150887.66	1588.40
#pos (%)	8 (25)	13 (40)	15 (47)	12 (38)
				
*Mann–Whitney U-test* h				
*P*-value NC *vs* M0	0.44	0.033	0.51	0.13
*P*-value NC *vs* M+	0.047	<0.0001	0.04	0.012
*P*-value M0 *vs* M+	0.15	0.002	0.004	0.002

BM=bone marrow; CK-19=cytokeratin-19; MAM=mammaglobin; PB=peripheral blood; RT–PCR=reverse transcription–polymerase chain reaction.

a Normalised relative gene expression of cytokeratin-19 mRNA in peripheral blood.

b Normalised relative gene expression of mammaglobin mRNA in peripheral blood.

c Normalised relative gene expression of cytokeratin-19 mRNA in bone marrow.

d Normalised relative gene expression of mammaglobin mRNA in bone marrow.

e Negative control patients.

f Patients with primary breast cancer without overt metastasis.

g Patients with metastatic breast cancer.

h Mann–Whitney *U*-test: differences in CK-19 or MAM mRNA expression in peripheral blood or bone marrow between the different subgroups of patients with breast cancer.

**Table 2:** Comparison between blood and BM samples for the detection of disseminated epithelial cells according to the relative gene expression of CK-19 and/ or MAM

**Number of patients**	**CK-19**	**MAM**
PB+BM+	8	11
PB+BM−	14	18
PB−BM+	34	19
PB−BM−	92	100
Concordance (%)	100/148 (68%)	111/148 (75%)

BM=bone marrow; CK-19=cytokeratin-19; MAM=mammaglobin; PB=peripheral blood.

**Table 3:** Relation of DEC in blood and bone marrow of patients with operable breast cancer with clinicopathological parameters

	**All patients**		**PB CK-19+patients**			**PB MAM+patients**			**BM CK-19+patients**			**BM MAM+patients**		
**Characteristic**	**No.**	**%**	**No.**	**%**	***P***	**No.**	**%**	***P***	**No.**	**%**	***P***	**No.**	**%**	***P***
*Menopausal status*					0.744			0.775			0.298			0.436
Pre	37	25.5	5	13.5		8	21.6		8	21.6		6	16.2	
Post	108	74.5	17	15.7		21	19.4		33	30.6		24	22.2	
Unknown	3													
														
*M status*					0.069			**0.001**			**0.009**			**0.006**
M0	116	78.4	14	12.1		16	13.8		27	23.3		18	15.5	
M+	32	21.6	8	25.0		13	40.6		15	46.9		12	37.5	
														
*Lymph node status*					0.49			0.661			0.183			0.314
N0	51	34.5	9	17.6		11	21.6		11	21.6		8	15.7	
N+	97	65.5	13	13.4		18	18.6		31	32.0		22	22.7	
														
*Tumour status*					0.611			0.981			0.718			0.444
T1T2	88	59.5	12	13.6		17	19.3		24	27.3		16	18.2	
T3T4	60	40.5	10	16.7		12	20.0		18	30.0		14	23.3	
														
*Histology*					0.126			0.144			0.627			0.642
Ductal	125	84.5	21	16.8		27	21.6		36	28.8		25	20.0	
Lobular	21	14.2	1	4.8		2	9.5		6	28.6		4	19.0	
Other	2	1.4	0	0.0		0	0.0		0	0.0		1	50.0	
														
*Histological grade*					0.051			0.44			0.34			0.118
I	35	24.3	2	5.7		5	14.3		10	28.6		3	8.6	
II	63	43.8	10	15.9		14	22.2		14	22.2		16	25.4	
III	46	31.9	10	21.7		10	21.7		17	37.0		11	23.9	
Unknown	4													
														
*ER status*					0.291			0.354			0.056			0.787
Negative	46	31.3	9	19.6		7	15.2		18	39.1		10	21.7	
Positive	101	68.7	13	12.9		22	21.8		24	23.8		20	19.8	
Unknown	1													
														
*PR status*					0.158			0.717			**0.028**			**0.026**
Negative	67	45.3	13	19.4		14	20.9		25	37.3		19	28.4	
Positive	81	54.7	9	11.1		15	18.5		17	21.0		11	13.6	
														
*Her2 expression*					**0.001**			0.221			0.459			0.085
Negative	126	86.3	14	11.1		23	18.3		34	27.0		23	18.3	
Positive	20	13.7	8	40.0		6	30.0		7	35.0		7	35.0	
Unknown	2													

BM=bone marrow; CK-19=cytokeratin-19; DEC=disseminated epithelial cells; ER=oestrogen receptor; MAM=mammaglobin; PB=peripheral blood; PR=progesterone receptor.

Statistically significant relations are present in bold.

**Table 4:** Results of univariate analysis (Cox's regression) for overall survival in patients with breast cancer

	**All patients**		
	**No of patients who died/total no.**	**Relative risk of death (95% CI)**	***P*-value**
*BM CK-19*			
+	11/42	3.26 (1.38–7.73)	0.007
−	10/106	1	
			
*BM MAM*			
+	7/30	2.72 (1.10–6.75)	0.031
−	14/118	1	
			
*BM MAM and CK*			
+/+	6/14	6.15 (2.16–17.45)	0.001
+/− or −/+	6/44	1.68 (0.6–4.73)	0.325
−/−	9/90	1	
			
*PB CK-19*			
+	3/22	1.45 (0.43–4.94)	0.553
−	18/126	1	
			
*PB MAM*			
+	5/29	1.65 (0.60–4.52)	0.334
−	16/119	1	
			
*PB MAM and CK*			
+/+	2/9	4.04 (0.9–18.11)	0.068
+/− or −/+	4/33	0.96 (0.32–2.91)	0.948
−/−	15/106	1	

BM=bone marrow; CI=confidence interval; CK-19=cytokeratin-19; MAM=mammaglobin.

**Table 5:** Results of multivariate analysis (Cox's regression) for overall survival in 148 patients with breast cancer (only variables significant on multivariate analysis are shown)

***n*=148**	**Risk ratio**	***P***	**95% CI**
Metastatic stage	6.31	<0.0001	2.40–16.57
BM CK-19 mRNA expression	3.60	0.009	1.39–9.37
Histological grade	3.02	0.004	1.43–6.38

BM=bone marrow; CI=confidence interval; CK-19=cytokeratin-19; MAM=mammaglobin.

**Table 6:** Comparison of tumour cell detection in blood samples and in bone marrow

**Ref.**	**Method**	**Number of patients**	**BM+/PB+**	**BM+/BM−**	**BM/PB+**	**BM−/PB−**	**Concordance (%)**
Schoenfeld *et al* (1997)	RT–PCR qualitative	65	8	15	7	35	66
(Schoenfeld *et al* (1997)	ICC	75	1	13	3	58	79
Berois *et al* (2000)	RT–PCR qualitative	37	10	9	3	15	68
(Stathopoulou *et al* (2002)	RT–PCR qualitative	73	30	19	0	24	74
Ismail *et al* (2004)	RT–PCR quantitative	47	22	14	3	8	64
Pierga *et al* (2004)	ICC	114	26	41	2	45	62

BM=bone marrow; ICC=immunocytochemical; MAM=mammaglobin; PB=peripheral blood; RT–PCR=reverse transcription–polymerase chain reaction.

## References

[bib1] Aerts J, Wynendaele W, Paridaens R, Christiaens MR, van den BW, van Oosterom AT, Vandekerckhove F (2001) A real-time quantitative reverse transcriptase polymerase chain reaction (RT–PCR) to detect breast carcinoma cells in peripheral blood. Ann Oncol 12: 39–46 11249047 10.1023/a:1008317512253

[bib2] Bartek J, Bartkova J, Schneider J, Taylor-Papadimitriou J, Kovarik J, Rejthar A (1986) Expression of monoclonal antibody-defined epitopes of keratin 19 in human tumours and cultured cells. Eur J Cancer Clin Oncol 22: 1441–1452 2439341 10.1016/0277-5379(86)90077-5

[bib3] Benoy IH, Elst H, Van dA I, Laere SV, Dam PV, Marck EV, Scharpe S, Vermeulen PB, Dirix LY (2004) Real-time RT–PCR correlates with immunocytochemistry for the detection of disseminated epithelial cells in bone marrow aspirates of patients with breast cancer. Br J Cancer 91: 1813–1820 15505629 10.1038/sj.bjc.6602189 PMC2410046

[bib4] Berois N, Varangot M, Aizen B, Estrugo R, Zarantonelli L, Fernandez P, Krygier G, Simonet F, Barrios E, Muse I, Osinaga E (2000) Molecular detection of cancer cells in bone marrow and peripheral blood of patients with operable breast cancer. Comparison of CK19, MUC1 and CEA using RT–PCR. Eur J Cancer 36: 717–723 10762743 10.1016/s0959-8049(99)00338-x

[bib5] Braun S, Pantel K (1998) Prognostic significance of micrometastatic bone marrow involvement. Breast Cancer Res Treat 52: 201–216 10066083 10.1023/a:1006164914610

[bib6] Braun S, Pantel K, Muller P, Janni W, Hepp F, Kentenich CR, Gastroph S, Wischnik A, Dimpfl T, Kindermann G, Riethmuller G, Schlimok G (2000) Cytokeratin-positive cells in the bone marrow and survival of patients with stage I, II, or III breast cancer. N Engl J Med 342: 525–533 10684910 10.1056/NEJM200002243420801

[bib7] Braun S, Vogl FD, Janni W, Marth C, Schlimok G, Pantel K (2003) Evaluation of bone marrow in breast cancer patients: prediction of clinical outcome and response to therapy. Breast 12: 397–404 14659112 10.1016/s0960-9776(03)00143-7

[bib8] Braun S, Vogl FD, Naume B, Janni W, Osborne MP, Coombes RC, Schlimok G, Diel IJ, Gerber B, Gebauer G, Pierga JY, Marth C, Oruzio D, Wiedswang G, Solomayer EF, Kundt G, Strobl B, Fehm T, Wong GY, Bliss J, Vincent-Salomon A, Pantel K (2005) A pooled analysis of bone marrow micrometastasis in breast cancer. N Eng J Med 353(8): 793–802 10.1056/NEJMoa050434 16120859

[bib9] Braun S, Vogl FD, Pantel K (2005) Disseminated tumor cells (DTC) in bone marrow (BM) and clinical outcome: final results of pooled analysis on 10-year survival of 4703 breast cancer patients. J Clin Oncol 23, (abstract no. 502)

[bib10] Corradini P, Voena C, Astolfi M, Delloro S, Pilotti S, Arrigoni G, Bregni M, Pileri A, Gianni AM (2001) Maspin and mammaglobin genes are specific markers for RT–PCR detection of minimal residual disease in patients with breast cancer. Ann Oncol 12: 1693–1698 11843246 10.1023/a:1013573108945

[bib11] Cristofanilli M, Budd GT, Ellis MJ, Stopeck A, Matera J, Miller MC, Reuben JM, Doyle GV, Allard WJ, Terstappen LWMM, Hayes DF (2004) Circulating tumor cells, disease progression, and survival in metastatic breast cancer. N Engl J Med 351: 781–791 15317891 10.1056/NEJMoa040766

[bib12] Cristofanilli M, Hayes DF, Budd GT, Ellis MJ, Stopeck A, Reuben JM, Doyle GV, Matera J, Allard WJ, Miller MC, Fritsche HA, Hortobagyi GN, Terstappen LWMM (2005) Circulating tumor cells: a novel prognostic factor for newly diagnosed metastatic breast cancer. J Clin Oncol 23: 1420–1430 15735118 10.1200/JCO.2005.08.140

[bib13] Diel IJ, Kaufmann M, Costa SD, Holle R, von Minckwitz G, Solomayer EF, Kaul S, Bastert G (1996) Micrometastatic breast cancer cells in bone marrow at primary surgery: prognostic value in comparison with nodal status. J Natl Cancer Inst 88: 1652–1658 8931609 10.1093/jnci/88.22.1652

[bib14] Gebauer G, Fehm T, Merkle E, Beck EP, Lang N, Jager W (2001) Epithelial cells in bone marrow of breast cancer patients at time of primary surgery: clinical outcome during long-term follow-up. J Clin Oncol 19: 3669–3674 11504748 10.1200/JCO.2001.19.16.3669

[bib15] Gerber B, Krause A, Muller H, Richter D, Reimer T, Makovitzky J, Herrnring C, Jeschke U, Kundt G, Friese K (2001) Simultaneous immunohistochemical detection of tumor cells in lymph nodes and bone marrow aspirates in breast cancer and its correlation with other prognostic factors. J Clin Oncol 19: 960–971 11181658 10.1200/JCO.2001.19.4.960

[bib16] Goldhirsh A, Senn H-J (2003) Adjuvant therapy for breast cancer. In Oxford Textbook of Oncology, Souhami RL, Tannock I, Hohenberger P, Horiot JC (eds) pp 1745–1761. Oxford, UK: Oxford University Press

[bib17] Grunewald K, Haun M, Urbanek M, Fiegl M, Muller-Holzner E, Gunsilius E, Dunser M, Marth C, Gastl G (2000) Mammaglobin gene expression: a superior marker of breast cancer cells in peripheral blood in comparison to epidermal-growth-factor receptor and cytokeratin-19. Lab Invest 80: 1071–1077 10908152 10.1038/labinvest.3780112

[bib18] Ismail MS, Wynendaele W, Aerts JL, Paridaens R, Gaafar R, Shakankiry N, Khaled HM, Christiaens MR, Wildiers H, Omar S, Vandekerckhove P, van Oosterom AT (2004) Detection of micrometastatic disease and monitoring of perioperative tumor cell dissemination in primary operable breast cancer patients using real-time quantitative reverse Transcription-PCR. Clin Cancer Res 10: 196–201 14734470 10.1158/1078-0432.ccr-0515-2

[bib19] Janni W, Gastroph S, Hepp F, Kentenich C, Rjosk D, Schindlbeck C, Dimpfl T, Sommer H, Braun S (2000) Prognostic significance of an increased number of micrometastatic tumor cells in the bone marrow of patients with first recurrence of breast carcinoma. Cancer 88: 2252–2259 10820346 10.1002/(sici)1097-0142(20000515)88:10<2252::aid-cncr8>3.0.co;2-q

[bib20] Landis JR, Koch GG (1977) The measurement of observer agreement for categorical data. Biometrics 33: 159–174 843571

[bib21] Leone F, Perissinotto E, Viale A, Cavalloni G, Taraglio S, Capaldi A, Piacibello W, Torchio B, Aglietta M (2001) Detection of breast cancer cell contamination in leukapheresis product by real-time quantitative polymerase chain reaction. Bone Marrow Transplant 27: 517–523 11313686 10.1038/sj.bmt.1702815

[bib22] Lin YC, Chen SC, Hsueh S, Lo YF, Chow-Wu YH, Liaw IC, Cheng AJ (2003) Lack of correlation between expression of human mammaglobin mRNA in peripheral blood and known prognostic factors for breast cancer patients. Cancer Sci 94: 99–102 12708482 10.1111/j.1349-7006.2003.tb01359.x PMC11160282

[bib23] Livak KJ, Schmittgen TD (2001) Analysis of relative gene expression data using real-time quantitative PCR and the 2(−Delta Delta C(T)) Method. Methods 25: 402–408 11846609 10.1006/meth.2001.1262

[bib24] Pierga JY, Bonneton C, Vincent-Salomon A, de Cremoux P, Nos C, Blin N, Pouillart P, Thiery JP, Magdelenat H (2004) Clinical significance of immunocytochemical detection of tumor cells using digital microscopy in peripheral blood and bone marrow of breast cancer patients. Clin Cancer Res 10: 1392–1400 14977842 10.1158/1078-0432.ccr-0102-03

[bib25] Ring AE, Zabaglo L, Ormerod MG, Smith IE, Dowsett M (2005) Detection of circulating epithelial cells in the blood of patients with breast cancer: comparison of three techniques. Br J Cancer 92: 906–912 15714202 10.1038/sj.bjc.6602418 PMC2361897

[bib26] Rubens RD (1998) Bone metastases – the clinical problem. Eur J Cancer 34: 210–213 9741323 10.1016/s0959-8049(97)10128-9

[bib27] Schindlbeck C, Janni W, Shabani N, Rack B, Gerber B, Schmitt M, Harbeck N, Sommer H, Braun S, Friese K (2004) Comparative analysis between the HER2 status in primary breast cancer tissue and the detection of isolated tumor cells in the bone marrow. Breast Cancer Res Treat 87: 65–74 15377852 10.1023/B:BREA.0000041583.72269.e1

[bib28] Schoenfeld A, Kruger KH, Gomm J, Sinnett HD, Gazet JC, Sacks N, Bender HG, Luqmani Y, Coombes RC (1997) The detection of micrometastases in the peripheral blood and bone marrow of patients with breast cancer using immunohistochemistry and reverse transcriptase polymerase chain reaction for keratin 19. Eur J Cancer 33: 854–861 9291805 10.1016/s0959-8049(97)00014-2

[bib29] Shapiro CL, Recht A (2001) Side effects of adjuvant treatment of breast cancer. N Engl J Med 344: 1997–2008 11430330 10.1056/NEJM200106283442607

[bib30] Silva AL, Tome MJ, Correia AE, Passos-Coelho JL (2002) Human mammaglobin RT–PCR assay for detection of occult breast cancer cells in hematopoietic products. Ann Oncol 13: 422–429 11996474 10.1093/annonc/mdf107

[bib31] Singletary SE, Allred C, Ashley P, Bassett LW, Berry D, Bland KI, Borgen PI, Clark G, Edge SB, Hayes DF, Hughes LL, Hutter RV, Morrow M, Page DL, Recht A, Theriault RL, Thor A, Weaver DL, Wieand HS, Greene FL (2002) Revision of the American Joint Committee on Cancer staging system for breast cancer. J Clin Oncol 20: 3628–3636 12202663 10.1200/JCO.2002.02.026

[bib32] Slade MJ, Singh A, Smith BM, Tripuraneni G, Hall E, Peckitt C, Fox S, Graham H, Luchtenborg M, Sinnett HD, Cross NC, Coombes RC (2005) Persistence of bone marrow micrometastases in patients receiving adjuvant therapy for breast cancer: results at 4 years. Int J Cancer 114: 94–100 15523696 10.1002/ijc.20655

[bib33] Slade MJ, Smith BM, Sinnett HD, Cross NC, Coombes RC (1999) Quantitative polymerase chain reaction for the detection of micrometastases in patients with breast cancer. J Clin Oncol 17: 870–879 10071278 10.1200/JCO.1999.17.3.870

[bib34] Smith BM, Slade MJ, English J, Graham H, Luchtenborg M, Sinnett HD, Cross NC, Coombes RC (2000) Response of circulating tumor cells to systemic therapy in patients with metastatic breast cancer: comparison of quantitative polymerase chain reaction and immunocytochemical techniques. J Clin Oncol 18: 1432–1439 10735890 10.1200/JCO.2000.18.7.1432

[bib35] Stathopoulou A, Gizi A, Perraki M, Apostolaki S, Malamos N, Mavroudis D, Georgoulias V, Lianidou ES (2003) Real-time quantification of CK-19 mRNA-positive cells in peripheral blood of breast cancer patients using the Lightcycler system. Clin Cancer Res 9: 5145–5151 14613993

[bib36] Stathopoulou A, Vlachonikolis I, Mavroudis D, Perraki M, Kouroussis C, Apostolaki S, Malamos N, Kakolyris S, Kotsakis A, Xenidis N, Reppa D, Georgoulias V (2002) Molecular detection of cytokeratin-19-positive cells in the peripheral blood of patients with operable breast cancer: evaluation of their prognostic significance. J Clin Oncol 20: 3404–3412 12177100 10.1200/JCO.2002.08.135

[bib37] Suchy B, Austrup F, Driesel G, Eder C, Kusiak I, Uciechowski P, Grill HJ, Giesing M (2000) Detection of mammaglobin expressing cells in blood of breast cancer patients. Cancer Lett 158: 171–178 10960767 10.1016/s0304-3835(00)00520-6

[bib38] Weinschenker P, Soares HP, Clark O, Giglio AD (2004) Immunocytochemical detection of epithelial cells in the bone marrow of primary breast cancer patients: a meta-analysis. Breast Cancer Res Treat 87: 215–224 15528964 10.1007/s10549-004-8691-1

[bib39] Wiedswang G, Borgen E, Karesen R, Kvalheim G, Nesland JM, Qvist H, Schlichting E, Sauer T, Janbu J, Harbitz T, Naume B (2003) Detection of isolated tumor cells in bone marrow is an independent prognostic factor in breast cancer. J Clin Oncol 21: 3469–3478 12972522 10.1200/JCO.2003.02.009

[bib40] Wiedswang G, Borgen E, Schirmer C, Karesen R, Kvalheim G, Nesland JM, Naume B (2005) Comparison of the clinical significance of occult tumor cells in blood and bone marrow in breast cancer. Int J Cancer 2005 Nov 14; [Epub ahead of print] 10.1002/ijc.21576 16287086

[bib41] Zach O, Kasparu H, Krieger O, Hehenwarter W, Girschikofsky M, Lutz D (1999) Detection of circulating mammary carcinoma cells in the peripheral blood of breast cancer patients via a nested reverse transcriptase polymerase chain reaction assay for mammaglobin mRNA. J Clin Oncol 17: 2015–2019 10561252 10.1200/JCO.1999.17.7.2015

[bib42] Zehentner BK, Persing DH, Deme A, Toure P, Hawes SE, Brooks L, Feng Q, Hayes DC, Critichlow CW, Houghton RL, Kiviat NB (2004) Mammaglobin as a novel breast cancer biomarker: multigene reverse transcription-PCR assay and sandwich ELISA. Clin Chem 50: 2069–2076 15375015 10.1373/clinchem.2004.038687 PMC1482781

